# Automated Video‐Based Approach for the Diagnosis of Tourette Syndrome

**DOI:** 10.1002/mdc3.14158

**Published:** 2024-07-07

**Authors:** Ronja Schappert, Julius Verrel, Nele Sophie Brügge, Frédéric Li, Theresa Paulus, Leonie Becker, Tobias Bäumer, Christian Beste, Veit Roessner, Sebastian Fudickar, Alexander Münchau

**Affiliations:** ^1^ Institute of Systems Motor Science University of Lübeck Lübeck Germany; ^2^ Institute of Medical Informatics University of Lübeck Lübeck Germany; ^3^ German Research Center for Artificial Intelligence Lübeck Germany; ^4^ Department of Neurology University Medical Center Schleswig‐Holstein Lübeck Germany; ^5^ Department of Pediatrics University Medical Center Schleswig‐Holstein Lübeck Germany; ^6^ Lübeck Centre for Rare Diseases University Medical Center Schleswig‐Holstein Lübeck Germany; ^7^ Department of Child and Adolescent Psychiatry, Faculty of Medicine TU Dresden Dresden Germany; ^8^ Faculty of Medicine, University Neuropsychology Center TU Dresden Dresden Germany; ^9^ Cognitive Psychology, Faculty of Psychology Shandong Normal University Jinan China

**Keywords:** Tourette, video based, automated, tic detection

## Abstract

**Background:**

The occurrence of tics is the main basis for the diagnosis of Gilles de la Tourette syndrome (GTS). Video‐based tic assessments are time consuming.

**Objective:**

The aim was to assess the potential of automated video‐based tic detection for discriminating between videos of adults with GTS and healthy control (HC) participants.

**Methods:**

The quantity and temporal structure of automatically detected tics/extra movements in videos from adults with GTS (107 videos from 42 participants) and matched HCs were used to classify videos using cross‐validated logistic regression.

**Results:**

Videos were classified with high accuracy both from the quantity of tics (balanced accuracy of 87.9%) and the number of tic clusters (90.2%). Logistic regression prediction probability provides a graded measure of diagnostic confidence. Expert review of about 25% of lower‐confidence predictions could ensure an overall classification accuracy above 95%.

**Conclusions:**

Automated video‐based methods have a great potential to support quantitative assessment and clinical decision‐making in tic disorders.

Gilles de la Tourette syndrome (GTS) is a common neurodevelopmental disorder characterized by the presence of motor and vocal tics for at least 1 year with onset before the age of 18 years.^1^ Tic frequency and severity are often assessed from patient videos, for example, using the Rush video rating scale.^2^ However, manual rating of acquired video materials depends on trained clinical experts and is time consuming. This limits the routine use of quantitative video‐based measures in routine clinical practice and clinical intervention studies, even though they may allow a more valid assessment of tics from videos obtained under different conditions, including more naturalistic settings.^3^ Here, we assess the potential of an automated, video‐based analysis to discriminate between videos of adults with GTS and healthy controls (HC). An automated tic detection algorithm^4^ was applied to videos from adults with GTS and HCs obtained after the Rush video protocol^2^ to compute tic summary scores discriminating between the 2 groups. Spontaneously occurring extra movements are also frequent in HCs,^5^ potentially undermining the classification of GTS adults versus HCs based only on the number of movements. Therefore, both the quantity and temporal characteristics of tics (or extra movements) were used for clinical prediction using cross‐validated logistic regression. Moreover, we explored a “hybrid” approach to clinical decision‐making, in which lower‐confidence automated predictions are reviewed by a movement disorders expert to ensure a high overall classification accuracy.

## Patients and Methods

### Participants

The clinical classification analysis was carried out on a dataset of videos from 42 adults with clinically diagnosed GTS (16 women; mean age: 29.1 years, range: 18–59 years; 107 videos) and individually matched HCs (16 women; 28.3 years, range: 18–58 years; 107 videos). Details of video recordings and the matching procedure are provided in the Supplemental Methods. Video recordings were obtained from the University of Lübeck, approved by the local ethics committee, after written informed consent. Clinical diagnoses were made by experienced neurologists according to the *Diagnostic and Statistical Manual of Mental Disorders, Fifth Edition* (DSM‐5), criteria^1^ based on clinical interview and personal examination. In addition, the Yale Global Tic Severity Scale (YGTSS^6^) was assessed in GTS participants (mean YGTSS: 44.5, range: 13–81). HCs reported no diagnosis of GTS or any other movement disorder.

### Machine Learning for Clinical Prediction

**FIG. 1:**
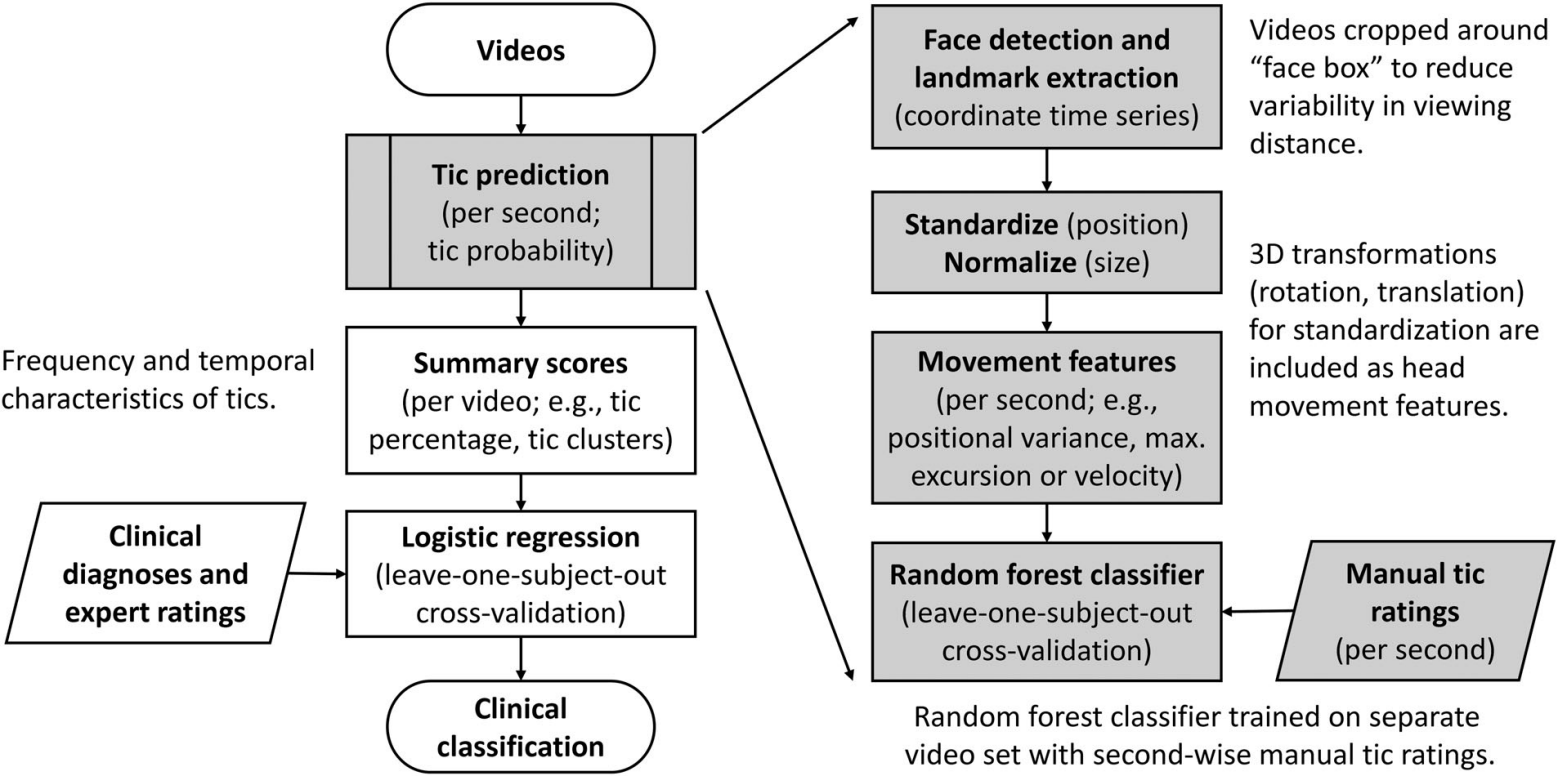
Video processing and machine‐learning pipeline. Right‐hand side: preprocessing and random forest classifier for tic detection. Left‐hand side: summary scores describing the amount and temporal characteristics of predicted tics were used for clinical classification (HC [healthy control] vs. GTS [Gilles de la Tourette syndrome]) by logistic regression.

The previously published tic detection algorithm^4^ (Fig. 1, right‐hand side) was slightly adapted to increase robustness with regard to variation in recording conditions between videos and within‐person positional variability (Supplemental Methods and Fig. S1). Subsequently, it was applied to a separate set of videos from the Rush protocol to compute tic summary scores, characterizing the quantity and temporal characteristics of predicted tics (Fig. 1, left‐hand side). These summary scores were (1) the proportion of “tic intervals” (%), (2) the mean tic probability, (3) the maximal duration of contiguous tic segments (seconds), (4) the maximal duration of tic‐free segments (seconds), and (5) the number of “tic clusters” per minute (Supplemental Materials and Fig. S2).

The ability of these tic summary scores to discriminate between GTS and HC participant videos was assessed using descriptive statistics, receiver operating characteristics (ROC), and univariate and multivariate logistic regression. To avoid biases due to class imbalance and to preserve group matching, classification performance on videos from each participant was evaluated using cross‐validation, that is, from a logistic regression model trained after excluding all videos from that participant as well as his or her individually matched participant. Classification performance was quantified using the balanced accuracy (arithmetic mean of sensitivity and specificity) and the area under the ROC curve (AUROC).

## Results

### Tic Summary Scores and Clinical Prediction

**FIG. 2:**
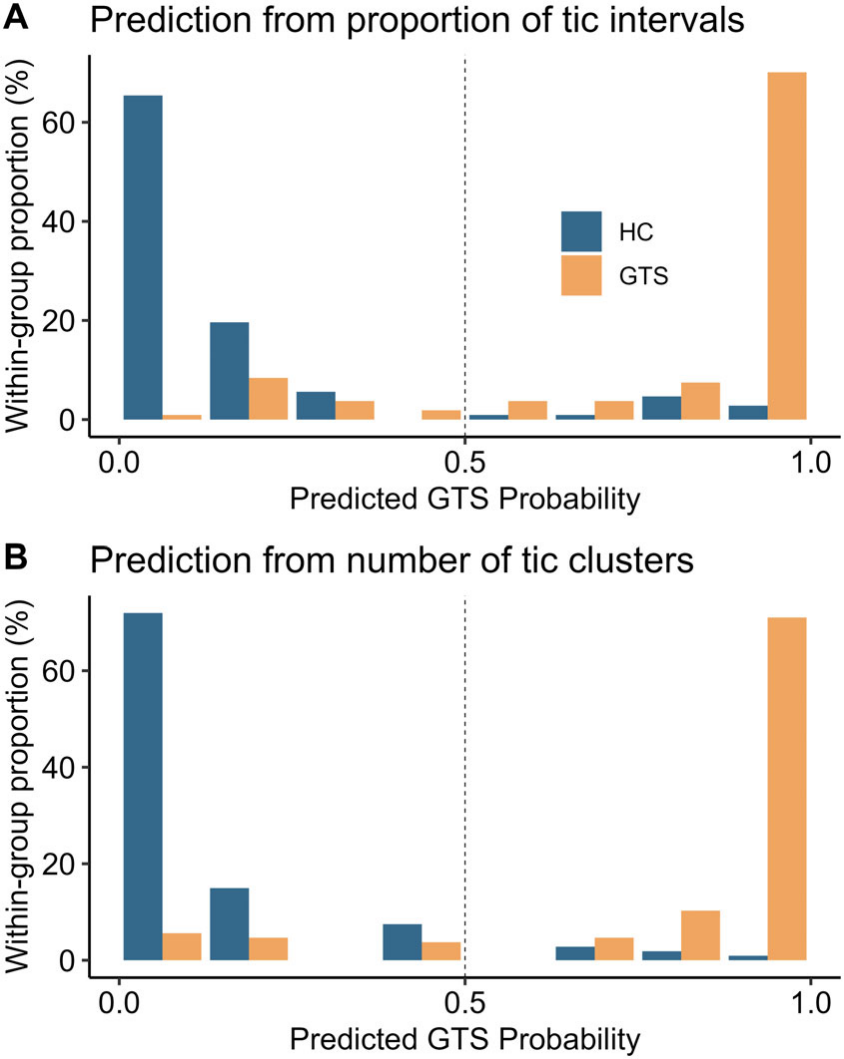
Distributions of clinical prediction probabilities from cross‐validated logistic regression based on (**A**) the proportion of tic intervals (1‐second intervals with “tic present”) and (**B**) the number of tic clusters per minute. HC, healthy controls; GTS, Gilles de la Tourette syndrome.

ROC and cross‐validated univariate logistic regression analyses (Table S1) suggest the number of tic clusters (AUROC: 0.95, balanced accuracy: 90.2%) and proportion of tic intervals (AUROC: 0.97, balanced accuracy: 87.9%) as the most promising clinical predictors. Per‐group distributions of these 2 tic summary scores are shown in Figure S3, and predicted GTS probabilities are shown in Figure 2. Multivariate logistic regression models did not improve classification performance (Supplemental Results). Both the number of tic clusters and the proportion of tic intervals correlated highly with manual tic counts (Supplemental Results and Fig. S4).

### Expert Review of Low‐Confidence Predictions

Video‐based clinical predictions from logistic regression include a probability of a video being from a GTS versus a HC participant (Fig. 2), and high‐confidence predictions for either group were associated with substantially higher classification accuracies compared to lower‐confidence predictions. For instance, predictions with a probability >0.9 in favor of either group had a classification accuracy of 97.9% for the proportion of tic intervals and 95.6% for the number of tic clusters. The corresponding accuracies for lower‐confidence predictions (prediction probability <0.9) were 67.6% for tic intervals and 74.1% for tic clusters.

This suggests a hybrid decision‐making approach, in which an expert review of lower‐confidence predictions ensures a high overall classification accuracy. For the current data set, the scenario described earlier (high‐confidence probability threshold of 0.9) would require reviewing 71 of 214 videos (33.2%) using the proportion of tic intervals as predictor, and 54 of 214 videos (25.2%) for the number of clusters. Assuming an expert accuracy of at least 95%, this would, in both cases, ensure an overall balanced accuracy above 95%.

## Discussion

We assessed the potential of video‐based, automated tic detection for discriminating between videos of adults with GTS and HCs. Cross‐validated logistic regression analyses indicate that clinical status can be predicted with high accuracy from the amount and temporal characteristics of automatically detected tics (or extra movements). Complementing high‐confidence predictions from this automated analysis with an expert review of lower‐confidence predictions would allow achieving overall accuracy scores above 95%.

Five tic summary scores were assessed for their ability to predict clinical status. Of these, the number of “tic clusters” (at least 3 consecutive 1‐second intervals with predicted tic) showed the highest classification performance, with a balanced accuracy above 90%. This is consistent with the clinical observation that tics often appear to cluster in time.^7^ We note, however, that temporal characteristics do not seem to be crucial to clinical prediction in the present analyses, as the proportion of 1‐second intervals with prediction “tic present” reached a comparable prediction accuracy (87%). Moreover, using manual tic counts from the Rush protocol, which do not take into account temporal characteristics either, allowed a correct classification of more than 90% of videos.

Logistic regression can not only be used for binary classification but also provides a prediction probability for each alternative (GTS vs. HC), that is, a graded measure of diagnostic confidence that could be particularly valuable to objectively assess treatment response in both routine clinical practice and clinical trials. Moreover, it opens the possibility of a “hybrid” diagnostic approach in which high‐confidence model‐based predictions are complemented by an expert review of lower‐confidence predictions. For the present dataset, such an approach would allow reaching 95% overall diagnostic accuracy while requiring manual screening of only a quarter to a third of all videos. This also addresses a general issue with the automated approach: as the video‐based prediction depends on overt symptom manifestation, GTS in persons with very subtle or infrequent tics will be less likely detected. However, these predictions will often be associated with a lower‐confidence prediction and could therefore be identified by the clinical expert in the proposed hybrid approach.

Supporting clinical decisions by machine‐learning methods raises both ethical and data protection issues. Even if our analysis indicates that 95% overall accuracy can be reached by reviewing only lower‐confidence predictions, the ultimate diagnostic decisions need to remain with a human expert. A machine‐learning‐based classification could save valuable time but carries the risk of overlooking diagnostic factors not taken into account by automated procedures. Conversely, abstaining from this support might render objective video‐based measures in larger‐scale clinical studies unfeasible. Concerning data protection, the applied algorithms are freely available, open‐source solutions that can be set up locally, require limited computational resources, and therefore do not require transfer to a central data processing site. In fact, the algorithms could even be implemented on mobile devices, with the potential to optimize recruitment procedures for clinical studies and advancing both clinical diagnostics and fundamental research by obtaining (preprocessed, de‐identified) data from more naturalistic settings and over longer periods of time, for example, at home while watching a movie. Previous research indicates that this may enable a more representative characterization of the amount and variety of tics of individual participants.^3^, ^8^

Our current approach takes into consideration only tics involving face and head movement. This is a limitation, as tics can occur at any limb across the entire body. Considering the rostro‐caudal distribution of tics,^9^ our approach probably captures a substantial proportion of tic behavior and may be sufficient for reliable clinical characterization in most cases. Although there are fewer tics in the lower body, there are also fewer additional movements in HCs. Therefore, the inclusion of lower‐body movements holds promise for improving classification. Future studies should address this limitation by implementing video‐based methods taking into account upper‐limb and whole‐body movements, as well as vocal tics.

In accordance with the Rush protocol,^2^ participants were instructed to stay calmly seated and not to perform intentional actions such as speaking, checking the phone, or eating. It remains therefore unclear whether the present analysis actually discriminates between tics and non‐tic movements or whether it mainly assesses a person's ability to suppress unwanted movements. This could be assessed by extending the present approach to more naturalistic settings, in which participants do not refrain from performing voluntary actions. Also, future research should use temporally more fine‐grained and multivariate analyses to assess how to best discriminate between intentional (or other non‐tic) movements and tics, as well as between tics and other abnormal movements such as functional tics, myoclonus, or stereotypies, which remains a challenge in clinical practice.

To summarize, the present machine‐learning approach allows predicting clinical status with high accuracy from summary scores characterizing the quantity and temporal characteristics of tics (or extra movements). In particular, “tic clusters” demonstrated high classification accuracy of about 90%. Logistic regression allows computing diagnostic confidence measures, supporting a “hybrid” diagnostic approach, in which lower‐confidence automated predictions are reviewed by clinical experts.

## Author Roles

(1) Research project: A. Conception, B. Organization, C. Execution; (2) Statistical analysis: A. Design, B. Execution, C. Review and critique; (3) Manuscript preparation: A. Writing of the first draft, B. Review and critique.

R.S.: 1B, 1C, 2A, 2B, 2C, 3B

J.V.: 1A, 1B, 1C, 2A, 2B, 2C, 3A, 3B

N.S.B.: 2A, 2C, 3B

F.L.: 2A, 2C, 3B

T.P.: 1B, 1C, 3B

L.B.: 1C, 3B

T.B.: 1A, 3B

C.B.: 3B

V.R.: 3B

S.F.: 2A, 2C, 3B

A.M.: 1A, 2A, 2C, 3B

## Disclosures

**Ethical Compliance Statement:** This research was approved by the Ethics Committee of the University of Lübeck, Germany. Written informed consent was obtained prior to participation. We confirm that we have read the journal's position on issues involved in ethical publication and affirm that this work is consistent with those guidelines.

**Funding Sources and Conflicts of Interest:** This work was funded by the German Research Foundation (DFG FOR 2698). The authors declare that there are no conflicts of interest relevant to this work.

**Financial Disclosures for the Previous 12 Months:** Alexander Münchau reports consultancies for PTC Therapeutics; is on the advisory board of the German Tourette Syndrome Association and the Alliance of Patients with chronic rare diseases; received honoraria from Desitin, Teva, and Takeda; received grants or financial support from Possehl‐Stiftung (Lübeck, Germany), Margot und Jürgen Wessel Stiftung (Lübeck, Germany), Tourette Syndrome Association (Germany), Interessenverband Tourette Syndrom (Germany), CHDI, Damp‐Stiftung (Kiel, Germany), Deutsche Forschungsgemeinschaft (DFG): projects 1692/3‐1, 4‐1, SFB 936, and FOR 2698 (project numbers: 396914663, 396577296, and 396474989), and European Reference Network—Rare Neurological Diseases (ERN—RND, project ID: 739510); is employed by the University of Lübeck, University Medical Center Schleswig‐Holstein, Campus Lübeck; receives royalties for the book *Neurogenetics* (Oxford University Press); and reports Commercial research support from Pharm Allergan, Ipsen, Merz Pharmaceuticals, and Actelion. Christian Beste is employed by TU Dresden, Universitätsklinikum Carl Gustav Carus, and receives grants from Deutsche Forschungsgemeinschaft, EKFS, and Volkswagen Stiftung. Frédéric Li is employed by the University of Lübeck (BMBF grant: 13GW0444E). Julius Verrel, Ronja Schappert, Leonie Becker, and Theresa Paulus are employed by the University Medical Center Schleswig‐Holstein, Campus Lübeck. Sebastian Fudickar reports stock ownership in medically related fields (MSCI World and potentially others); reports grants from BMBF Germany LaOLA Project, Forschungspool University Oldenburg, Germany, and Alexander von Humboldt Society; and is employed by the University of Lübeck. Tobias Bäumer reports consultancies for Pelzerhaken Children's Centre; is employed at the University Hospital Schleswig Holstein; and receives honoraria from Pelzerhaken Children's Centre, Allergan/AbbVie, Ipsen Pharma, and Merz Therapeutics. Veit Roessner is employed by the University Medical Center Carl Gustav Carus an der Technischen Universität Dresden and receives royalties from Hogrefe. Nele Sophie Brügge is employed by the German Research Center for Artificial Intelligence.

## Supporting information


**Data S1.** Supplemental methods and results.
**Figure S1.** Video processing and facial landmark detection. (**A**) Original videos were cropped (larger rectangle) to a multiple (150% to each side, 50% above, and 100% below) of the face region (smaller rectangle) determined using automated face detection (MediaPipe BlazeFace). (**B**) From the cropped video, three‐dimensional (3D) positions of 468 facial landmarks as well as estimated 3D face/head position and orientation were extracted using MediaPipe FaceMesh. (**C**) Facial landmarks were (3D‐) rotated to a standard frontal position and spatially normalized (mean: 0, standard deviation: 1). Video processing algorithms are from the MediaPipe framework.
**Figure S2.** Per‐second tic probabilities predicted using the random forest tic detection algorithm, for 2 representative participants with Gilles de la Tourette syndrome (GTS; **A**: few tics, **C**: many tics) and healthy controls (HC; **B** and **D**). The threshold for tic detection was 0.5 (dashed horizontal line). The duration of video segments for clinical prediction was 2.5 minutes (150 seconds).
**Figure S3.** Distributions for 2 tic summary scores for the 2 groups, healthy controls (HC), and people with Gilles de la Tourette syndrome (GTS). (**A**) Proportion of tic intervals (1‐second intervals with predicted “tic present”). (**B**) Number of tic clusters (consisting of at least 3 1‐second intervals) per minute.
**Figure S4.** Scatter plot illustrating the correlation between the proportion of tic intervals from the automated analysis (1‐second intervals with random forest‐ prediction “tic present”) and the manually rated tic frequency from the Rush protocol.
**Table S1.** Descriptive statistics and classification performance for tic summary scores.

## Data Availability

Original video data cannot be published for data protection reasons. Data and R code for the statistical analysis are available from a public data repository (https://osf.io/46vxm/). The Python code for video preprocessing and tic detection is available upon request.
