# Supplementary material for: Automated Video‐Based Approach for the Diagnosis of Tourette Syndrome
Source: Mov Disord Clin Pract. 2024 Jul 7;11(9):1136–40. doi: 10.1002/mdc3.14158 (PMC11452802; doi:10.1002/mdc3.14158)
Supplement: Supplementary file 1 — Data S1. Supplemental methods and results. Figure S1. Video processing and facial landmark detection. (A) Original videos were cropped (larger rectangle) to a multiple (150% to each side, 50% above, and 100% below) of the face region (smaller rectangle) determined using automated face detection (MediaPipe BlazeFace). (B) From the cropped video, three‐dimensional (3D) positions of 468 facial landmarks as well as estimated 3D face/head position and orientation were extracted using MediaPipe FaceMesh. (C) Facial landmarks were (3D‐) rotated to a standard frontal position and spatially normalized (mean: 0, standard deviation: 1). Video processing algorithms are from the MediaPipe framework. Figure S2. Per‐second tic probabilities predicted using the random forest tic detection algorithm, for 2 representative participants with Gilles de la Tourette syndrome (GTS; A: few tics, C: many tics) and healthy controls (HC; B and D). The threshold for tic detection was 0.5 (dashed horizontal line). The duration of video segments for clinical prediction was 2.5 minutes (150 seconds). Figure S3. Distributions for 2 tic summary scores for the 2 groups, healthy controls (HC), and people with Gilles de la Tourette syndrome (GTS). (A) Proportion of tic intervals (1‐second intervals with predicted “tic present”). (B) Number of tic clusters (consisting of at least 3 1‐second intervals) per minute. Figure S4. Scatter plot illustrating the correlation between the proportion of tic intervals from the automated analysis (1‐second intervals with random forest‐ prediction “tic present”) and the manually rated tic frequency from the Rush protocol. Table S1. Descriptive statistics and classification performance for tic summary scores. [file MDC3-11-1136-s001.pdf]

Supplemental Materials

Schappert, Verrel et al. Automated video-based approach for the diagnosis of Tourette syndrome

Supplemental Methods

Videos for second-wise tic detection

Videos with second-wise manual tic ratings were used to train the second-wise tic prediction algorithm using a random forest (RF) classifier[1]. These videos were acquired in the context of a recent study investigating the temporal relationship between premonitory sensations ("urges") and tics[2]. Participants were seated at a desk in front of a computer screen, continuously indicating the intensity of their subjective urge using the computer mouse. Upper body video recordings were acquired using a webcam (Logitech c930e, 30 Hz, 1920x1080 pixels) mounted on top of the screen. Five-minute videos from this paradigm were rated per second by two trained raters for the presence/absence and intensity (0: no tic, intensity: 1-8) of upper-body motor and vocal tics, as well as body region of motor tics (face, head/neck, trunk, arm/hand), using a video annotation tool [3]. Only motor tic ratings indicating a tic in the face or head/neck region were used for the present analysis. The inter-rater reliability (Pearson correlation) for tic presence/absence was 0.84 on average (range 0.67-0.94). Disagreements regarding tic presence were reviewed and resolved by a movement disorders expert (A.M.). Motor tic intensity ratings were averaged between raters. Further details have been reported before[2].

Twenty-one videos with tic ratings from 21 participants with clinically diagnosed GTS (10 female; mean age 31.6 years, age range 18-52 years) were available for the RF training. Videos from four participants (two with high and two with low number of tics) were selected as a validation set for determining optimal model hyperparameters. The RF classifier was evaluated on the remaining 17 participants, using leave-one-out cross-validation.

Random forest classifier for second-wise tic detection

The previously published ML-based approach for tic detection[1] was slightly adapted to increase robustness with regard to variation in recording conditions between videos and within-person positional variability (Manuscript Figure 1, right-hand side). Video sampling rates were standardized to 25 Hz prior to utilizing the MediaPipe framework[4] for face localization and head/facial motion estimation. Videos were cropped to a rectangle defined by the average face position and size, determined for each video using automatic face detection (MediaPipe BlazeFace; Figure S1). Three-dimensional (3D) positions of 468 facial landmarks as well as estimated 3D face/head position and orientation were extracted using MediaPipe FaceMesh. To standardize face motion data within and across participants, facial landmark coordinates were rotated and moved to a default frontal position, spatially normalized (to mean 0 and standard deviation 1; see Figure S1), and temporally low-pass filtered for noise reduction (3-point median filter). The resulting facial landmark data as well as head orientation and position estimates

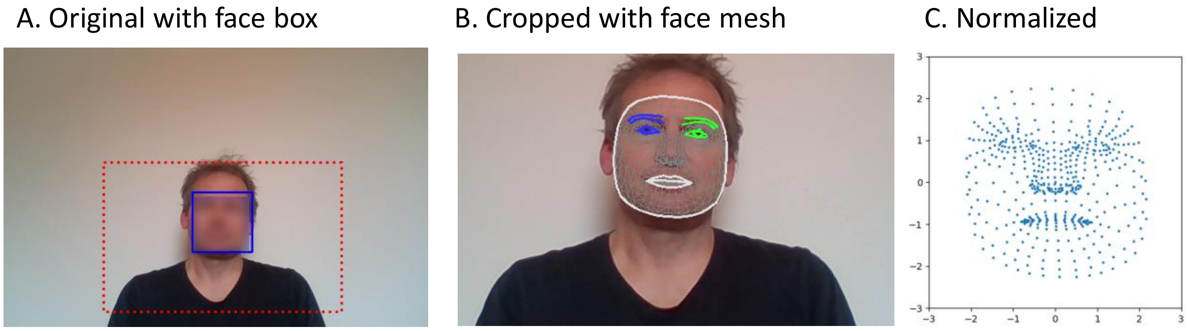

Figure S1. Video processing and facial landmark detection. A. Original videos were cropped (larger rectangle) to a multiple (150% to each side, 50% above, 100% below) of the face region (smaller rectangle) determined using automated face detection (MediaPipe BlazeFace). B. From the cropped video, 3D positions of 468 facial landmarks as well as estimated 3D face/head position and orientation were extracted using MediaPipe FaceMesh. C. Facial landmarks were (3D-) rotated to a standard frontal position and spatially normalized (mean 0 and standard deviation 1). Video processing algorithms are from the the MediaPipe framework[4].

were used to compute several movement features (twelve for each coordinate) characterizing movement amplitude and speed within 1-second intervals: the total positive (negative) movement, i.e., sum of positive (negative) frame-to-frame changes along a particular movement axis; maximal continuous positive (negative) movement, i.e., maximal sum of consecutive positive (negative) frame-to-frame changes; maximal continuous positive (negative) movement, divided by the duration of the corresponding time interval; maximal positive (negative) frame-to-frame change; average positive (negative) frame-to-frame change; total movement range (maximum minus minimum); variance. For the frontal plane, positive/negative movement directions are specified by the orientations of the X and Y axis (see Figure S1). For the anterior-posterior axis, positive means “out of the paper” (or screen).

Using manual per-second motor tic ratings, a RF classifier was trained to predict presence/absence of tics in 1-second intervals. We previously found tic detection performance to be highest when restricted to tics with a rated intensity ≥ 3, defined as “mild but clearly noticeable” tics[1] and therefore used the same threshold here.

Hyperparameters for the RF classifier were optimized on a separate validation set consisting of four videos from people with GTS (two with high and two with low number of tics and tic severity, using the tree-structured Parzen Estimator (100 epochs) from the Python Optuna toolbox [5]. Based on this, we used a RF classifier with 715 trees, each with a maximum depth of 5, and a minimum of 20 samples for every leaf. Tic detection performance was evaluated on the remaining 17 videos using leave-one-out cross-validation. To maximize the available information for clinical prediction (described below), the “final” RF classifier applied to Rush videos was trained on the entire labeled data set (N=21, i.e., including validation videos).

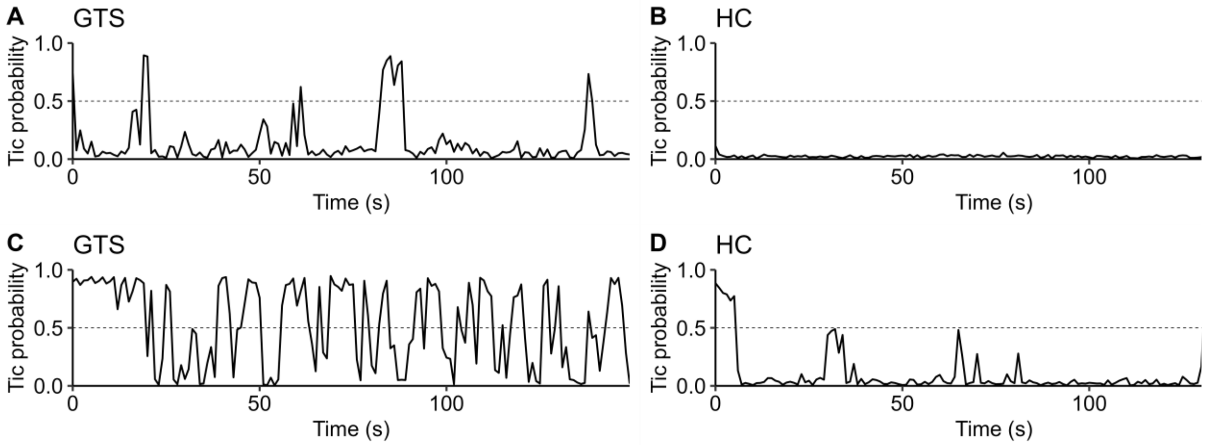

Figure S2. Per-second tic probabilities predicted by the random forest tic detection algorithm, for two representative participants with Gilles de la Tourette Syndrome (GTS; A: few tics, C: many tics) and healthy controls (HC; B and D). The threshold for tic detection was 0.5 (dashed horizontal line). The duration of video segments for clinical prediction was 2.5 minutes (150s).

In addition to a binary prediction (tic present / absent), the proportion of trees in the RF indicating "tic present" can be interpreted as a tic probability for each 1s-interval. Figure S2 shows exemplary tic probability time series from a GTS and a HC participant.

Videos for clinical classification

Videos from the Rush video protocol [6] acquired in the context of three studies[2,7] were used to train, validate and evaluate the ability to predict clinical status based on second-wise data (tic present/absent) from the RF classifier. Following the Rush protocol, participants were comfortably seated in a chair in front of a camcorder (different cameras and settings, 25-50 Hz, 1920x1080 pixels) and instructed to face the camera, stay calmly seated, and not to perform intentional actions such as speaking, checking the phone or eating/chewing [6]. Recordings had a duration of 10 minutes, divided in four conditions (2 x 2 design: experimenter absent/present, full-body/close-up view). As our automated tic detection is based on facial and head motion, only the 2.5-minute interval with the close-up view with experimenter absent was used for automated tic detection. Besides clinical information for each participant (GTS vs. HC), the (manually determined) tic counts per minute and the Rush total score are available for most of these video segments.

Participants contributing videos with second-wise tic ratings used to train the RF classifier were excluded to avoid any bias resulting from using videos from the same participants for tic detection training and clinical classification. As the total number of available videos and participants from the HC group (218 videos from 92 participants) exceeded that of the GTS group (119 videos from 42 participants), participants with GTS were individually matched to HC based on age, sex, and the number of contributed videos[8] in order to minimize potential confounding group differences and ensure equal numbers of participants and videos per groups, This resulted in a perfect matching of the two groups (42 participants per group) regarding sex, a mean absolute age deviation of 1.5 years (range 0-7.5 years) and a close matching for the number of videos (mean absolute difference 0.35, range 0-2). For each matching pair, the same number of videos was included in the analysis.

Tic summary scores

We computed five tic summary scores, characterizing the amount and temporal characteristics of predicted tics:

1) The **proportion of "tic intervals":** The proportion (%) of 1s-intervals within the 2.5-minute recording, for which the automated RF-based tic prediction indicated "tic present".

2) The **mean tic probability**: The average across all 1s-intervals of the 2.5-minute recording of per-second tic probabilities (range 0.0-1.0) indicated by the RF-predictor. The tic probability corresponds to the number of "trees" in the RF indicating "tic present".

3) The **maximal duration of contiguous tic segments**: The maximal duration (in seconds) of consecutive 1s-intervals for which the RF-based tic detection indicated "tic present".

4) The **maximal duration of tic-free segments:** The maximal duration (in seconds) of consecutive 1s-intervals for which the RF-based tic detection indicated "tic absent".

5) The **number of "tic clusters" per minute:** A tic cluster was defined as a group of three or more consecutive 1s-intervals, for which the RF-based tic detection indicated "tic present". The number of tic clusters was counted in the 2.5-minute recording and normalized to 1 minute (by dividing by 2.5).

Supplemental Results

Second-wise tic detection

The automated second-wise tic detection (Figure 1, right-hand side) showed comparable performance to our previous publication[1] for correctly predicting the presence/absence of a tic in a given 1-second interval, with an average balanced accuracy per video of 0.79 (range 0.57-0.93; sensitivity: 0.14-0.94, specificity: 0.68-1.00, AUROC: 0.79-0.99). This confirms that the adaptations of the video processing procedure, introduced to increase robustness against variation in recording conditions expected in videos from clinical settings, did not negatively affect tic detection performance.

Tic summary scores and clinical prediction

Summary statistics and clinical prediction performance (GTS vs. HC) for the five tic summary scores are shown in Table S1. Per-group distributions for the two summary scores best discriminating between GTS and HC are plotted in Figure S3.

Table S1. Descriptive statistics and classification performance for tic summary scores.

Predictor	HC[1]	GTS[1]	AUROC	Sensitivity	Specificity	Balanced Accuracy
Number of tic clusters (per minute)	0.47 ± 0.50 (0.0-2.4)	2.96 ± 1.33 (0.0-6.0)	0.95	86.0%	94.4%	90.2%
Proportion of tic intervals (%)	7.07 ± 8.24 (0.0-40.7)	46.79 ± 23.04 (7.3-100.0)	0.97	85.0%	90.7%	87.9%
Mean tic probability (0-1)	0.11 ± 0.08 (0.0-0.4)	0.45 ± 0.18 (0.1-0.9)	0.97	86.0%	87.9%	86.9%
Max. duration of tic-free intervals (seconds)	101.55 ± 42.84 (15.0-150.0)	23.96 ± 19.72 (0.0-99.0)	0.95	91.6%	82.2%	86.9%
Max. duration of tic intervals (seconds)	4.41 ± 3.43 0.0-25.0)	18.64 ± 19.60 (2.0-150.0)	0.92	79.4%	84.1%	81.8%

Note. Summary scores are reported as mean ± standard deviation and range. Sensitivity, specificity and balanced accuracy are based on cross-validated logistic regression analysis. Predictors (rows) are arranged by decreasing classification performance (balanced accuracy). *Abbreviations:* HC = Healthy controls. GTS = Gilles de la Tourette Syndrome. AUROC = Area under the receiver operating characteristic (ROC) curve.

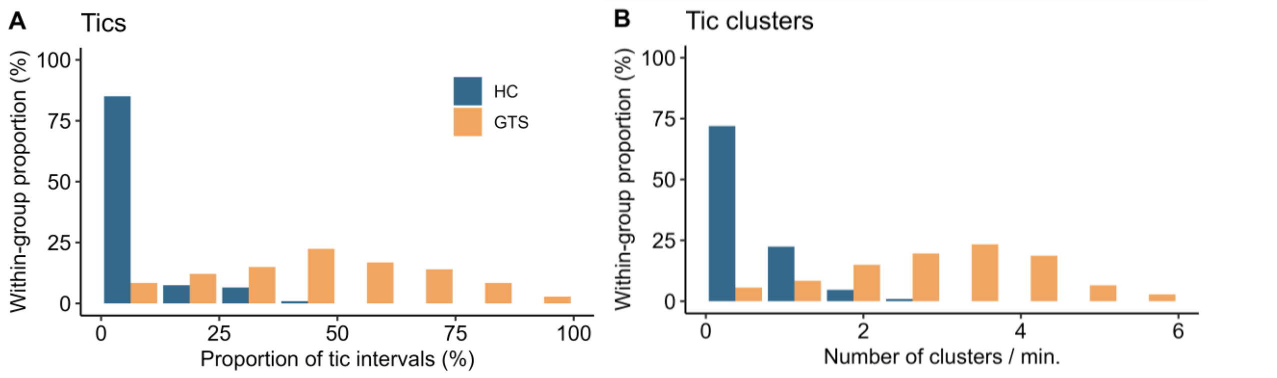

Figure S3. Distributions for two tic summary scores for the two groups, healthy controls (HC) and people with Gilles de la Tourette (GTS). A. Proportion of tic intervals (1s-intervals with predicted "tic present"). B. Number of tic clusters (consisting of at least three 1s-intervals) per minute.

Multivariate logistic regression for clinical prediction

Multivariate logistic regression with all five summary scores as predictors indicated the number of tic clusters ($\chi^2(1)=16.5$, $p<0.001$), maximal tic-free duration (χ^2 (1)=5.53, $p=0.019$) and maximal tic duration (χ^2 (1)=4.13, p=0.042) as significant predictors. However, the corresponding cross-validated multivariate logistic regression analyses did not indicate an advantage of including either all summary scores (balanced accuracy 89.7%) or only the three significant predictors (balanced accuracy 89.3%), compared to the univariate logistic regression using only the number of tic clusters reported above (90.2%, see Table S1).

Comparison of automated measures to manual tic counts

Manual tic counts (from the Rush video protocol) were available for 64 (87) of the 107 GTS (HC) videos included in the main analysis. To compare automated (proportion of 1s-intervals with RF-prediction “tic present”) to manual tic counts while preserving comparability of the two groups, we created a data set of 64 videos from each group, matched for participant age and sex [8]. The proportion of tic intervals correlated highly with manual tic counts (Figure S4), both across groups (r=0.84, p<0.001) and within each group (GTS: r=0.58, p<0.001; HC: r=0.68, p<0.001). This was also the case for the number of tic clusters (correlation across groups: r=0.80, p<0.001; GTS: r=0.43, p<0.001, HC: r=0.63, p<0.001).

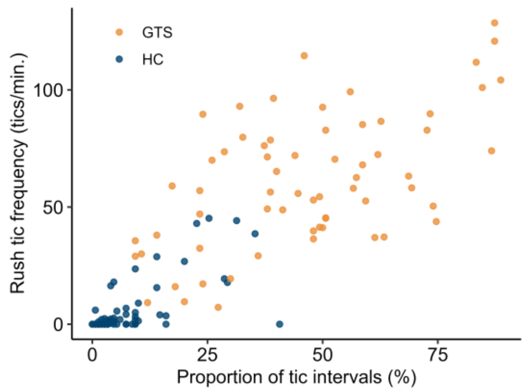

Figure S4. Scatter plot illustrating the correlation between the proportion of tic intervals from the automated analysis (1s-intervals with RF-prediction “tic present”) and the manually rated tic frequency from the Rush protocol.

Cross-validated logistic regression on this subset of videos showed comparable classification performance for manual tic counts (balanced accuracy 90.6%) and the automated measures proportion of tic intervals (88.3%) and number of tic clusters (91.4%).

References

1. Brügge NS, Sallandt GM, Schappert R, et al. Automated Motor Tic Detection: A Machine Learning Approach. Mov Disord 2023;38(7):1327–1335.

2. Schubert L, Verrel J, Behm A, et al. Inter-individual differences in urge-tic associations in Tourette syndrome. Cortex 2021;143:80–91.

3. DataVyu Team. Datavyu: A Video Coding Tool. Databrary Project [Internet]. 2014;Available from: https://datavyu.org/

4. Lugaresi C, Tang J, Nash H, et al. Mediapipe: A framework for perceiving and processing reality. In: Third workshop on computer vision for AR/VR at IEEE computer vision and pattern recognition (CVPR). 2019

5. Akiba T, Sano S, Yanase T, et al. Optuna: A Next-generation Hyperparameter Optimization Framework [Internet]. In: Proceedings of the 25th ACM SIGKDD International Conference on Knowledge Discovery & Data Mining. New York, NY, USA: Association for Computing Machinery; 2019 p. 2623–2631.[cited 2024 Jan 15] Available from: https://dl.acm.org/doi/10.1145/3292500.3330701

6. Goetz CG, Pappert EJ, Louis ED, et al. Advantages of a modified scoring method for the rush video-based tic rating scale. Movement Disorders 1999;14(3):502–506.

7. Paulus T, Wernecke L, Lundie A, et al. The Role of the Left Inferior Parietal Cortex in Gilles de la Tourette Syndrome—An rTMS Study. Biomedicines 2023;11(3):980.

8. Ho D, Imai K, King G, Stuart EA. MatchIt: Nonparametric Preprocessing for Parametric Causal Inference. Journal of Statistical Software 2011;42:1–28.
